# Exploring the Potential Roles of Band 3 and Aquaporin-1 in Blood CO_2_ Transport–Inspired by Comparative Studies of Glycophorin B-A-B Hybrid Protein GP.Mur

**DOI:** 10.3389/fphys.2018.00733

**Published:** 2018-06-19

**Authors:** Kate Hsu

**Affiliations:** Transfusion Medicine and Immunogenetics Laboratories, MacKay Memorial Hospital, Tamsui, Taiwan

**Keywords:** band 3, aquaporin-1, CO_2_ transport, erythrocytes, metabolon, glycophorin, GP.Mur

## Abstract

The Cl^—^/HCO_3_^—^ exchanger band 3 is functionally relevant to blood CO_2_ transport. Band 3 is the most abundant membrane protein in human red blood cells (RBCs). Our understanding of its physiological functions mainly came from clinical cases associated with band 3 mutations. Severe reduction in band 3 expression affects blood HCO_3_^—^/CO_2_ metabolism. What could happen physiologically if band 3 expression is elevated instead? In some areas of Southeast Asia, about 1–10% of the populations express GP.Mur, a glycophorin B-A-B hybrid membrane protein important in the field of transfusion medicine. GP.Mur functions to promote band 3 expression, and GP.Mur red cells can be deemed as a naturally occurred model for higher band 3 expression. This review first compares the functional consequences of band 3 at different levels, and suggests a critical role of band 3 in postnatal CO_2_ respiration. The second part of the review explores the transport of water, which is the other substrate for intra-erythrocytic CO_2_/HCO_3_^—^ conversion (an essential step in blood CO_2_ transport). Despite that water is considered unlimited physiologically, it is unclear whether water channel aquaporin-1 (AQP1) abundantly expressed in RBCs is functionally involved in CO_2_ transport. Research in this area is complicated by the fact that the H_2_O/CO_2_-transporting function of AQP1 is replaceable by other erythrocyte channels/transporters (e.g., UT-B/GLUT1 for H_2_O; RhAG for CO_2_). Recently, using carbonic anhydrase II (CAII)-filled erythrocyte vesicles, AQP1 has been demonstrated to transport water for the CAII-mediated reaction, CO_2(g)_ + H_2_O ⇌ HCO_3_^—^_(aq)_ + H^+^_(aq)_. AQP1 is structurally associated with some population of band 3 complexes on the erythrocyte membrane in an osmotically responsive fashion. The current findings reveal transient interaction among components within the band 3-central, CO_2_-transport metabolon (AQP1, band 3, CAII and deoxygenated hemoglobin). Their dynamic interaction is envisioned to facilitate blood CO_2_ respiration, in the presence of constantly changing osmotic and hemodynamic stresses during circulation.

## Introduction

Band 3 (SLC4A1), a membrane protein of 911 amino acids, belongs to the SLC4A family of HCO_3_^—^ (and CO_3_^2-^) transporters (Choi, 2012; Romero et al., 2013). Band 3 is also known as anion exchanger (AE1), as band 3-mediated HCO_3_^—^ transport is coupled at a 1:1 ratio with an anti-parallel flux of Cl^—^, the most abundant anion physiologically. A truncated isoform of AE1, which lacks the first 65 amino acids of band 3 or erythroid AE1, is expressed in the acid-secreting intercalated cells of the kidney (Kollert-Jons et al., 1993). Band 3 is the most abundant membrane protein in human erythrocytes (1–1.2 million molecules per RBC), with two major functions: (1) cell mechanical support through its physical linkage to ankyrin and the cytoskeletal network (Low et al., 1991); (2) blood CO_2_/HCO_3_^—^ exchange through its bicarbonate transport activity. The latter impacts acid-base homeostasis in various human physiological systems. This review first explores the function of band 3 in blood CO_2_ exchange based on previous studies using cellular/animal models with different band 3 levels. In my laboratory, this was achieved experimentally by comparing RBCs expressing an unusual surface antigen named GP.Mur, to RBCs devoid of this antigen. GP.Mur RBCs generally express 20% or more band 3, and can be used as an experimental model for higher band 3 expression in erythrocytes (Hsu et al., 2009, 2011).

## Clues From Early Gp.Mur Proteomic Research

**FIGURE 1:**
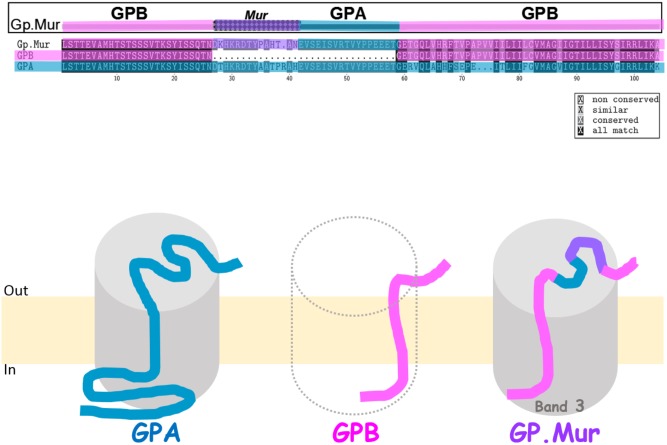
Glycophorin B-A-B hybrid protein GP.Mur interacts with band 3. **(Top)** Protein sequence alignment of GPA, GPB, and GP.Mur, which was first published in Blood (Hsu et al., 2009), is modified here with color coding. The sequences of GPA and GPB are color-coded with blue and pink, respectively. The antigenic Mur peptide at the cross-over region in GP.Mur is color-coded purple. The configuration of glycophorin B-A-B is revealed by the combination of pink-(purple)-blue-pink colors. **(Bottom)** GPA, GPB, and GP.Mur are homologous membrane proteins, each with a single transmembrane span. Their *N*-terminal sequences are located extracellular, with heavy glycosylation (not shown); their *C*-terminal sequences are intracellular. Both GPA and GP.Mur interact with band 3 (gray cylinder), and GPB does not.

GP.Mur, commonly known as Miltenberger subtype III (Mi.III) in Southeast Asia, is an erythrocyte antigen of the MNS blood group system (Tippett et al., 1992; Lomas-Francis, 2011). The prevalence of GP.Mur is between 1 and 7% in regions of Southeast Asia including Taiwan, but very low in other parts of the world (Issitt, 1985; Broadberry and Lin, 1994; Hsu et al., 2013). GP.Mur structurally exhibits the configuration of glycophorin B-A-B (**Figure 1**). GP.Mur evolved from homologous gene recombination of *glycophorin B* and *glycophorin A*, and is essentially *glycophorin B* with a piece of *glycophorin A* inserted in the middle (**Figure 1**) (Huang and Blumenfeld, 1991; Hsu et al., 2015b). Alloantibodies against the antigenic Mur sequence at one of the two cross-over sites (e.g., anti-Mur; anti-Mi^a^) are naturally occurred in ∼0.5% local Taiwanese. So if a person bearing such an alloantibody is accidentally transfused with GP.Mur RBCs, an acute intravascular hemolytic reaction might occur (Lin and Broadberry, 1998). There are ∼1 × 10^6^ glycophorin A (GPA) protein molecules and 1.7–2.5 × 10^5^ glycophorin B (GPB) molecules in an average human RBC (Gardner et al., 1989). In people with heterozygous *GYP.Mur* (*GYP.Mur^het^*), GP.Mur replaces half of GPB protein expression; in people with homozygous *GYP.Mur* (*GYP.Mur^ho^*), GP.Mur substitutes all the expression of GPB.

The early proteomic work from my group identifies unique structural features of GP.Mur-associated protein complexes on the RBC membrane (“ghosts”). Importantly, GP.Mur red cells express significantly more band 3. The protein-protein interaction between band 3 and AQP1 on the GP.Mur RBC membrane is substantial, compared to that on the GP.Mur-negative cell membrane (Hsu et al., 2009). Band 3 and AQP1 were thought not to interact on the erythrocyte membrane (Cho et al., 1999).

Because the only known difference between GP.Mur and GP.Mur-negative erythrocytes is the inserted sequence from *glycophorin A* in *GYP.Mur* (*GYP.B-A-B*), we hypothesized that the *glycophorin A*-derived peptide in GP.Mur promoted the protein expression of band 3. We took the experimental approach similar to an early study done in *Xenopus* oocytes, which demonstrates increase of band 3 levels upon GPA co-expression (Groves and Tanner, 1992). We transfected band 3 alone, or together with GP.Mur (or GPA as the positive control) into HEK-293 mammalian cultured cells. We found that like GPA, GP.Mur also enhances band 3 co-expression in the heterologous expression system (Hsu et al., 2009). For comparison, GPB appears to lack the chaperone-like activity of GPA for band 3, and is unable to promote band 3 protein expression (Groves and Tanner, 1992, 1994). Thus, the 32-amino acid-long peptide inserted in GP.Mur, which GPB lacks, renders GP.Mur to be functionally equivalent to GPA in supporting band 3 expression (**Figure 1**) (Hsu et al., 2009).

The interaction between GPA and band 3 begins in the endoplasmic reticulum (ER) where GPA facilitates protein synthesis of band 3 (Groves and Tanner, 1992). GPA-band 3 complexes are found on the cell surface, but the two proteins also traffic independently to the plasma membrane (Pang and Reithmeier, 2009; Giger et al., 2016). This may explain why overall band 3 expression does not seem to be substantially affected by GPA-deficiency in individuals with the very rare blood types that lack GPA expression, i.e., En(a-), M^k^M^k^, and Mi.V (Bruce et al., 2004). On the other hand, in the RBCs with the rare En(a-), M^k^M^k^, and Mi.V phenotypes, the absence of GPA prolongs the retention time of band 3 in the ER and Golgi apparatus, resulting in excessive build-up of *N*-glycans in band 3 (Gahmberg et al., 1976; Bruce et al., 2004). Therefore, band 3 biosynthesis is slower without the chaperone-like activity of GPA. These structural features of GPA-deficient band 3 complexes are associated with their lower efficiencies in anion transport (e.g., for Cl^—^, I^—^, and sulfate^—^) (Bruce et al., 2004). By fluorescence polarization studies, GPA-deficient band 3 complexes on the erythrocyte membrane also exhibit higher degrees of rotational freedom (Bruce et al., 2004), though a much earlier measurement shows that GPA or its substantial sialic acid content does not drastically affect the rotational freedom of band 3 (Nigg et al., 1980). The discrepancy was recently rigorously explored by single particle tracking of separately labeled band 3 and GPA. That new work reveals that GPA-band 3 interaction could become transitory during physiological processes like band 3 phosphorylation (Giger et al., 2016).

## Band 3 as the Rate-Limiting Factor for Blood Co_2_ Respiration

In human, over two-thirds of CO_2_ metabolite from tissues are carried in the form of soluble bicarbonate until exhalation in the lungs. By utilizing CO_2(g)_/HCO_3_^—^_(aq)_ exchange, the capacity of one’s CO_2_ tolerance can be expanded substantially. This chemical conversion, CO_2(g)_ + H_2_O ⇌ HCO_3_^—^_(aq)_ + H^+^_(aq)_, is primarily facilitated by intraerythrocytic carbonic anhydrase II (CAII), as the rate of its spontaneous conversion outside RBCs (*t*_1/2_ = 14 s) is too slow to meet physiological demands (Reithmeier, 2001).

**FIGURE 2:**
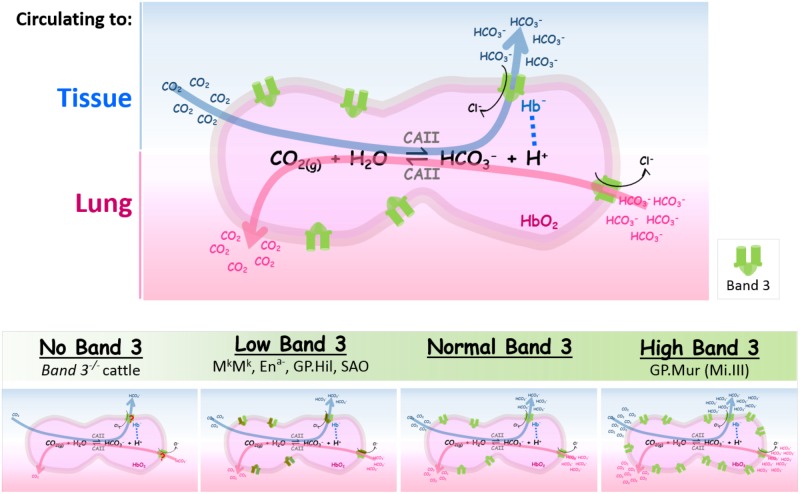
The expression levels of band 3 are directly correlated with the efficiencies of blood CO_2_/HCO_3_^—^ exchange. **(Top)** Intraerythrocytic CO_2_/HCO_3_^—^ exchange in tissues or in lung alveoli is summarized in the reversible chemical reaction catalyzed by CAII [CO_2(g)_ + H_2_O ⇌ HCO_3_^—^_(aq)_ + H^+^_(aq)_]. When RBCs circulate to systemic capillaries, tissue metabolite CO_2_ enters RBCs to be converted into HCO_3_^—^ by intraerythrocytic CAII. HCO_3_^—^, one of the two products from this forward reaction, exits from RBCs through band 3 (shown by the blue arrow which indicates the direction of the reaction in tissues). The direction of the counterion Cl^—^ flux through band 3 is indicated by a black arrow. Proton, the other product generated by the forward reaction, is absorbed by deoxy Hb^—^ that is transiently associated with band 3 (shown by a dotted line connecting Hb^—^ and H^+^). When RBCs circulate to the lungs, HCO_3_^—^ rushes into RBCs to be converted into CO_2_ for expiration (shown by the pink arrow which indicates the reverse direction of the reaction). Band 3 is symbolized as the green dimeric gate. **(Bottom)** The spectrum of band 3 expression levels in RBCs is outlined in the shaded green bar; their differential impacts on blood CO_2_ transport are illustrated in the four cartoon diagrams below. *The four bottom diagrams from left to right:* (1) Absence of band 3 reduces much HCO_3_^—^ transport across the RBC membrane, rendering CO_2_/HCO_3_^—^ exchange to be very inefficient. (2) Low band 3 expression can be found in some band 3/GPA mutations that result in semi-dysfunctional band 3 transporters, as described in the section of *“Low or no band 3 expression”* (shown here by the brown/green gate symbols for “structurally abnormal band 3”). (3) Normal band 3 expression. (4) Higher band 3 expression in GP.Mur RBCs (shown by more green gates on the cell membrane) increases the efficiency as well as the capacity of intraerythrocytic CO_2_/HCO_3_^—^ conversion and HCO_3_^—^ transport across the RBC membrane. The different numbers of CO_2_/HCO_3_^—^ in the bottom four diagrams represent schematically the different magnitudes of CO_2_/HCO_3_^—^ fluxes (metabolic flows) that correspond to the different levels of band 3 expression on the red cell membrane; they do not reflect extracellular concentrations of CO_2_ and HCO_3_^—^.

When blood circulates to the capillary bed, CO_2_ metabolite enters RBCs via diffusion and/or gas channels (e.g., AQP1 and RhAG) (Cooper and Boron, 1998; Nakhoul et al., 1998; Endeward et al., 2008; Musa-Aziz et al., 2009). Abundant CAII inside erythrocytes (10^6^ molecules/RBC) facilitates hydration of CO_2(g)_ to HCO_3_^—^_(aq)_. Bicarbonate permeates through band 3 dimers or dimerized dimers (tetramers), following its concentration gradient across the RBC membrane (Reithmeier et al., 2016). Because HCO_3_^—^ transport by band 3 utilizes Cl^—^ as the counter anion, band 3-mediated bicarbonate flux is electroneutral, and consumes very little or no energy. In the lung alveoli, CO_2_ expiration drives the above reaction toward the left (dehydration of bicarbonate). Extracellular bicarbonate needs to enter red cells via band 3 to be converted into CO_2(g)_ by intraerythrocytic CAII (**Figure 2**, top).

CAII and band 3 are structurally and functionally coupled during blood CO_2_ transport and respiration (Sowah and Casey, 2011). Carbonic anhydrase is the most efficient enzyme known today, with *K_cat_* or its turnover number (∼6 × 10^5^/sec) at least 10 times faster than the rate of Cl^—^/HCO_3_^—^ exchange of an erythroid AE1 molecule (∼5 × 10^4^ ions/sec) (Maren, 1967; Brahm, 1977). Since each human red cell expresses identical numbers of CAII and band 3 molecules (each ∼10^6^ molecules/RBC), the efficiency of CAII-catalyzed CO_2_/HCO_3_^—^ conversion is about an order higher than the rate of AE1-conducted Cl^—^/HCO_3_^—^ flux through the cell membrane. For comparison, the rate of water permeation through an AQP1 is ∼3 × 10^9^ H_2_O/sec. Intraerythrocytic water is used in CO_2_/HCO_3_^—^ exchange. With 160,000–200,000 AQP1 on the erythrocyte membrane, the rate of water transport via AQP1 is estimated 480–600 times faster than the enzymatic activity of intraerythrocytic CAII. Therefore, the anion exchange activity of erythroid AE1 is the rate-limiting step for blood CO_2_ transport (Reithmeier, 2001), which is directly related to the capacity of CO_2_ respiration in an individual (Hsu et al., 2015a).

## The Function of Band 3 in Respiratory Physiology

### Low or No Band 3 Expression

The respiratory support by erythroid AE1 is critical for human survival (Daniels, 2013). Up to date, there were only three clinical cases about the complete absence of band 3 protein expression resulted from deleterious homozygous mutations of band 3 (Ribeiro et al., 2000; Picard et al., 2014; Kager et al., 2015). These three patients between 3 and 4 years old at the time of publishing the case reports survive essentially by relying on regular transfusion. They were all delivered prematurely, because of fetal hydrops and severe anemia. One of them, later identified to be the only case of Southeast Asian Ovalocytosis (SAO) band 3 homozygosity in the world, was initially rescued by *in utero* transfusion at 22 weeks gestation, and then delivered at 29 weeks gestation (Picard et al., 2014). Intriguingly, besides apparent acidosis due to the absence of Cl^—^/HCO_3_^—^ transport, bone marrow analyses for the three patients revealed similarly marked dyserythropoiesis. In the homozygous SAO patient, his dyserythropoiesis is characterized by abnormally larger erythroblasts with two or more nuclei (Picard et al., 2014), which resonates the main features of band 3 knockout in zebrafish—erythroid-specific cytokinesis and dyserythropoietic anemia (Paw et al., 2003). Therefore, in addition to blood CO_2_ transport, band 3 is functionally essential for correct mitosis during erythropoiesis.

SAO is prevalent in certain ethnic groups in Southeast Asia and the Southwest Pacific (Kimura et al., 2003). Besides one case of SAO homozygosity survived by rigorous medical intervention as mentioned above (Picard et al., 2014), almost all SAO cases are resulted from heterozygous mutation of SAO band 3 (Daniels, 2013). SAO mutation is characterized by gene deletion in codons 400–408 of band 3 that corresponds to a location at the border between the cytoplasmic domain and the first transmembrane domain (Liu et al., 1990; Jarolim et al., 1991). The red cells from heterozygous SAO carriers express heterodimeric band 3 that is composed of a SAO band 3 subunit and a normal band 3 subunit. From transport kinetic studies, the anion transport efficiency of SAO heterozygotes is only half of the transport efficiency of normal cells (Jennings and Gosselink, 1995). Heterozygous SAO patients presumably survive with ∼50% efficiency of physiologic Cl^—^/HCO_3_^—^ exchange. For SAO patients comorbid with distal renal tubular acidosis (dRTA)-associated mutations in band 3, their band 3-mediated HCO_3_^—^ transport is further reduced to less than 5% of the transport efficiencies (Jarolim et al., 1991; Liu et al., 1994; Bruce et al., 2000).

Intriguingly, total deficiency of band 3 has been found in a breed of Japanese black cattle with a premature stop codon in the coding sequence of band 3, which corresponds to codon 646 in human band 3 (Inaba et al., 1996). Despite that few of these *band 3*^—^*^/^*^—^ cattle survive to adulthood, the survived ones present growth retardation, severe anemia, and pathophysiological conditions related to band 3 deficiencies, i.e., membrane instability-related spherocytosis, reduced Cl^—^/HCO_3_^—^ anion fluxes across the erythrocyte membrane, mild acidosis, and smaller capacities for blood CO_2_ transport (Inaba et al., 1996).

These phenotypes of band 3-deficient cattle could be recapitulated in the above-mentioned, transfusion-dependent human cases and in the neonates of *band 3*^—^*^/^*^—^ mice: growth retardation, spherocytosis, severe anemia, and substantially reduced capacities of anion transport by RBCs (Inaba et al., 1996; Peters et al., 1996; Southgate et al., 1996; Ribeiro et al., 2000; Picard et al., 2014; Kager et al., 2015). But unlike the naturally bred, band 3-deficient cattle found in Japan, band 3 knockout mice do not survive for more than 2 weeks postpartum. The proportion of the homozygous neonates generated was about one quarter, obeying the Mendel’s Laws of Inheritance (Southgate et al., 1996). Therefore, complete deficiency of band 3 does not affect the survival of *band 3*^—^*^/^*^—^ mice in the fetal stage, but postnatally. For human fetuses, blood CO_2_ transport primarily relies on CO_2_ and not HCO_3_^—^, due to restricted bicarbonate permeation through the placenta (Gabbe, 2012). Inferred from the findings in human, cattle and mouse, HCO_3_^—^ transporter band 3 becomes functionally critical for CO_2_ respiration after birth.

### High Band 3 Expression

On the other end of the spectrum of band 3 expression is GP.Mur. GP.Mur erythrocytes express significantly more band 3, and present phenotypes opposite to the phenotypes from band 3-deficient RBCs, i.e., superior membrane resilience toward stress and larger capacities for CO_2_ transport and pH buffering (Inaba et al., 1996; Peters et al., 1996; Southgate et al., 1996; Hsu et al., 2009). These functional advantages of GP.Mur RBCs due to high band 3 levels are expected. But could this explain the long-standing observation of superior physical endurance and athleticism prevalent in Taiwanese from the tribes with exceedingly high frequencies of GP.Mur phenotype (Hsu et al., 2009; Hsu, 2011)? To probe into the impacts of GP.Mur (or higher band 3 expression) at the systemic level, we conducted a large human study on respiratory physiology. We challenged the recruited healthy adults with “3-minute stepping exercise” (a standardized fitness test), which temporarily increased the demands for respiratory gas exchange. Respiratory parameters were measured before and right after the exercise challenge. Indeed, people with GP.Mur blood type breathed and exhaled CO_2_ significantly faster than those lacking this blood type (about 1 minute faster in clearance of CO_2_ generated from 3-minute exercise) (Hsu et al., 2015a). We did not observe significant differences between GP.Mur-positive and GP.Mur-negative groups in exercise-induced changes of heart rates, %O_2_ saturation, or lactate production.

We also noticed that the changes of blood CO_2_ and bicarbonate due to this mild exercise challenge were smaller in GP.Mur-positive subjects (Hsu et al., 2015a). One’s blood gas levels are tuned by his or her breathing rhythm, which is regulated by the respiratory centers, or the central controller neurons located in medulla and pons of the brainstem (West, 2005). Since the efficiencies of CO_2_ respiration are differentiable by GP.Mur phenotype, the neurological responses from the brainstem respiratory centers are expected to be differentiable (Hsu et al., 2015a).

**Figure 2** summarizes the impacts of differential band 3 expression on blood CO_2_ transport. At one extreme with little or no band 3 expression, the lack of erythroid AE1 appears lethal for most mammalian species, except a breed of Japanese cattle who could grow to adulthood with severe systemic defects (Inaba et al., 1996). The red cell membrane from band 3-deficient animals is very unstable and fragile, resulting in spherocytosis and severe anemia. Band 3 knockout substantially dissipates most of the DIDS-sensitive anion transport, resulting in acidosis and reduced anion exchange and CO_2_ transport (Inaba et al., 1996; Peters et al., 1996; Southgate et al., 1996). The other end of the spectrum is represented by GP.Mur, an example of higher band 3 expression. The higher densities of band 3 in GP.Mur erythrocytes strengthen membrane stability, and enlarge the capacity of anion exchange. Higher band 3 expression manifests systemically in more efficient clearance of CO_2_ (Hsu et al., 2009, 2015a). Perhaps not coincidentally, several Taiwanese tribes with highest rates of GP.Mur prevalence in the world (20–90% GP.Mur-positive) have long been recognized for superior physical performance, endurance, and even athleticism, compared to other ethnic groups in Taiwan (1–2% GP.Mur-positive) (Shikakogi 1912/1985; Wu and Hu, 1999; Aoki, 2002; Kuo, 2007; Lai, 2013).

## Osmotically Sensitive Interaction Between AQP1 and Band 3

Mammalian CO_2_ transport relies on constant hydration of CO_2_ and dehydration of HCO_3_^—^ inside erythrocytes. Approximately 0.1% of unbound, intraerythrocytic water is estimated to be used for CO_2_/HCO_3_^—^ conversion, after excluding maximally 85% of intraerythrocytic water that is structured or bound H_2_O (0.34–1.44 g structured H_2_O/g dry mass in intact erythrocytes) (Cameron et al., 1988). Intraerythrocytic H_2_O can be considered as an unlimited substrate. But unexpectedly, our early study found significantly more AQP1-band 3 protein complexes in GP.Mur-positive than in GP.Mur-negative RBCs, despite that the protein levels of AQP1 are the same in both GP.Mur-positive and GP.Mur-negative erythrocytes (Hsu et al., 2009). Does this suggest that the functionality of AQP1 is associated with the functionality of band 3? Or is this AQP1-band 3 interaction unique to GP.Mur-expressed cells?

We later employed a sensitive, biophysical approach—fluorescence resonance energy transfer by fluorescence lifetime imaging microscopy (FLIM-FRET), to verify AQP1-band 3 interaction that was initially identified by proteomics (Hsu et al., 2009). FLIM measures the fluorescence lifetimes of single fluorophore molecules. FRET, a phenomenon that only occurs within the range of dipole–dipole interaction (10 nm), can be measured by FLIM with much less bias than traditional intensity-based measurements. By this approach, AQP1 and band 3 are found at a distance of 8 nm from each other on the erythrocyte membrane. Though their interaction is more obvious in GP.Mur RBCs, AQP1-band 3 interaction also exists in RBCs lacking GP.Mur. Importantly, this AQP1-band 3 interaction could be dissipated by hypotonic conditioning (e.g., by diluting the physiological buffer HBSS with water to ∼250 mOsm/Kg) (Hsu et al., 2017). This osmotically sensitive interaction between AQP1 and band 3 conceivably allows erythrocytes to sense and respond to hemodynamic shear stress, as well as changes of erythrocyte volume and shape during circulation. Thus, AQP1-band 3 interaction on the red cell membrane is dynamic, not static. The protein-protein interaction between GPA and band 3 is also dynamic and changeable by essential cellular processes, such as phosphorylation of band 3 (Giger et al., 2016).

## Revisit the Model—“Co_2_-Transport Metabolon”

Band 3, CAII, and AQP1 are all functionally associated with one another for intraerythrocytic CO_2_/HCO_3_^—^ conversion. After the discovery of the protein-protein interaction between CAII and band 3 (Vince and Reithmeier, 2000; Sterling et al., 2001a,b), the band 3-CAII protein complex has been proposed to be the core of a CO_2_-transport metabolon because of their structural-functional correlation (Reithmeier, 2001; Bruce et al., 2003; Sowah and Casey, 2011). Recently, AQP1 has also been demonstrated to be spatially associated with CAII, and to transport H_2_O for CAII-mediated catalysis (Vilas et al., 2015). Nonetheless, the existence of a CO_2_-transport metabolon was challenged in two studies, where the protein-protein interaction between band 3 and CAII could not be identified either by a tsA201 (SV40-transformed HEK) heterologous expression system, or by the direct binding assay with individual purified proteins (Piermarini et al., 2007; Al-Samir et al., 2013).

Perhaps the interactions between band 3 and other red cell proteins are much more complicated than we initially envisioned. Band 3 protein complexes are currently categorized into three types, with differences in their individual components and in their distinct cellular localization relative to the submembranous spectrin network. About half of band 3 molecules are anchored submembranously onto specific sites of the spectrin network in dimeric forms (junctional complexes) and tetrameric forms (ankyrin complexes); the other half of band 3 molecules (∼640,000 copies/RBC) are mobile dimers located within the corrals set by the submembranous spectrin mesh (Steck, 1978; Burton and Bruce, 2011). AQP1 is confined to the corrals of the underlying cytoskeletal network (Cho et al., 1999). Conceivably, membrane-bound, mobile AQP1 and cytosolic CAII may preferentially interact with one or more types of the band 3 complexes, resulting in structurally and functionally differentiable complexes on the erythrocyte membrane. Very likely the population of cytoskeleton-independent band 3 preferentially interacts with AQP1, as the protein-protein interaction between AQP1 and band 3 was measured by FLIM-FRET using inside-out vesicles (IOVs) from erythrocyte membrane that were depleted of much spectrin cytoskeleton and non-integral membrane-bound proteins (Hsu et al., 2017). Additionally, as AQP1-band 3 interaction is adaptable to osmotic changes (Hsu et al., 2017), it is possible that the interaction between CAII and band 3 could also be transitory and sensitive to osmotic or hemodynamic shear stresses (Lazaro et al., 2014).

## Inferred From Osmotically Sensitive Aqp1-Band 3 Interaction: “Co_2_-H_2_O on Demand” in the Co_2_-Transport Metabolon

**FIGURE 3:**
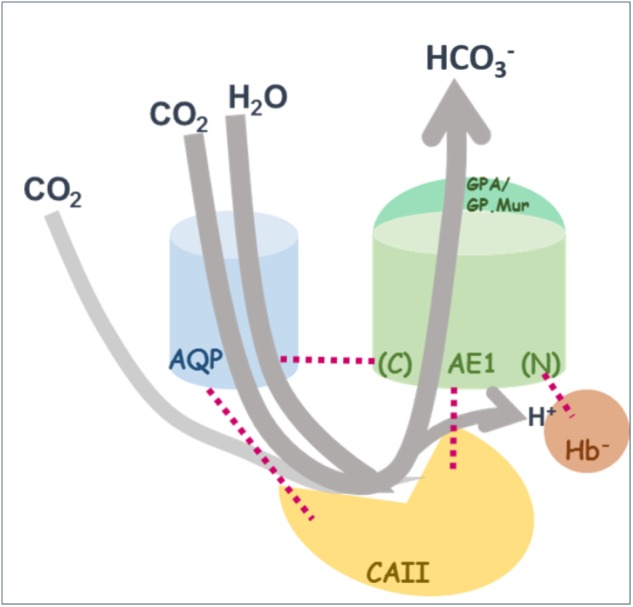
A revised model for the “CO_2_-transport metabolon.” As illustrated in the cartoon diagram, the CO_2_-transport metabolon can be deemed as a transiently extended CAII machinery, which is built upon transient interactions among the four proteins: AQP1, erythroid AE1, CAII, and deoxy Hb^—^ (shown by red dotted lines). The *C*-terminal and *N*-terminal regions of AE1 are indicated by (*C*) and (*N*). Illustrated here in gray arrow lines is the direction of the conversion reaction in systemic capillaries: CO_2_ is converted into HCO_3_^—^, followed by export of bicarbonate and absorption of proton by Hb^—^.

The idea of a CO_2_-transport metabolon, first proposed by Reithmeier, is based on the experimental observation that the major proteins supporting this intraerythrocytic reaction (CO_2_ + H_2_O ⇌ HCO_3_^—^ + H^+^) are spatially adjacent to one another to maximize the efficiency of blood CO_2_ transport (**Figure 3**) (Reithmeier, 2001). This forward reaction takes place when red cells circulate to capillaries surrounded by tissues, where O_2_ fluxes out of RBCs and CO_2_ fluxes in. Deoxygenated hemoglobin (deoxy Hb) preferentially binds to the *N*-terminal, cytoplasmic domain of band 3 (Walder et al., 1984; Castagnola et al., 2010; Chu et al., 2016). The forward reaction is driven by the removal of the two products: (1) band 3-mediated export of HCO_3_^—^; (2) absorption of proton by negatively charged deoxy Hb which is transiently bound to band 3.

It is estimated that ∼60% of CO_2_ flux in or out of RBCs is via AQP1 gas channel. The rest of CO_2_ flux is likely through another gas channel, RhAG, and/or direct diffusion across the lipid bilayer (Blank and Ehmke, 2003; Endeward et al., 2006, 2008; Wang et al., 2007; Boron, 2010). The transient association between AQP1 and band 3/CAII/deoxy Hb conceivably enables the formation of a spatially connected passage for each step of CO_2_/HCO_3_^—^ exchange, when erythrocytes circulate to systemic capillaries: (1) the entry of the substrates CO_2_ and H_2_O into RBCs through AQP1, (2) intraerythrocytic hydration of CO_2_ by CAII, (3) the exit of one of the two reaction products, bicarbonate, from erythrocytes via band 3, and (4) the absorption of the other reaction product, proton, by nearby deoxy Hb^—^ (Chu et al., 2016) (**Figure 3**). This transient arrangement of having H_2_O/CO_2_ channel AQP1, adjacent to band 3, CAII and proton-absorbing deoxy Hb^—^, conceivably allows an almost uninterrupted “channeling” of reactant influx and product outflow (Moraes and Reithmeier, 2012). The transient structural coupling also allows CO_2_/HCO_3_^—^ exchange to be primarily carried out near the surface and the submembranous zone of erythrocytes whenever possible, which may further support the efficiency of CO_2_ transport.

From the enzymatic perspective, the active site of CAII is conically shaped and lined with ordered water molecules (Liang and Lipscomb, 1990). This network of ordered water molecules supports rapid proton transfer (Mikulski et al., 2013), which is rate-limiting for CAII-mediated catalysis. By supporting the formation of “low barrier H^+^-bond” (LBHB), the network of ordered water allows CO_2_ loosely bound to CAII (Krebs et al., 1993). This weak substrate (CO_2_) binding allows a nearly complete occupancy of CO_2_ at the active site, contributing to the ultra-high efficiency of CAII. Indeed, the concentration of CO_2_ at the active site of CAII is very high (0.45 M) (Krebs et al., 1993; Domsic et al., 2008). This implies that the CAII-extended machinery (or the channel built by transient linkages of AQP1, band 3, CAII, and deoxy Hb) could function with a mode of “CO_2_-H_2_O on demand,” since the two reaction substrates—CO_2_ and H_2_O, are always near complete saturation in the active site of CAII. Conceivably, this design of “CO_2_-H_2_O on demand” could increase the sensitivity of circulating erythrocytes to blood CO_2_ gradients, as even a very small change in the concentration of the substrate could trigger CAII-mediated catalysis.

## The Potential Role of AQP1 in Blood Co_2_ Respiration

AQP1 as a H_2_O/CO_2_ channel is functionally replaceable by other channels/transporters (Iserovich et al., 2002; Yang and Verkman, 2002; Zeuthen et al., 2016). The first clue is that AQP1 null people (of Colton blood types) appear healthy (Preston et al., 1994). This strongly hints that its H_2_O/CO_2_-transporting function might not be exclusively carried out physiologically by AQP1 alone. The only known symptom in AQP1 null people shows upon water deprivation; AQP1 deficiency reduces their capability to concentrate urine or reabsorb free water at the medullary collecting duct of the kidney (King et al., 2001). Similarly, AQP1-deficient mice show normal survival and reduced ability in urine concentration (Ma et al., 1998).

The second clue came from comparing the single knockouts of AQP1 or urea transporter UT-B to the double knockout of AQP1 and UT-B. The RBCs from AQP1 null mice exhibit significantly reduced osmotic H_2_O permeability. The RBCs from UT-B null mice are similarly water-permeable as the RBCs from wild-type mice. But water permeability in the RBCs of AQP1 and UT-B double-knockout mice is further reduced. Double knockout of AQP1 and UT-B show reduced survival and retarded growth, in addition to reduced urinary concentrating ability (Yang and Verkman, 2002). This suggests that urea transporter does not primarily function to permeate water, but it can transport water when needed. Another RBC membrane transporter—glucose transporter GLUT1, is also capable of permeating water (Iserovich et al., 2002; Zeuthen et al., 2016). Therefore, AQP1 is the main water channel in human (especially erythrocytes), but its function in water permeation can be alternatively supported by urea transporter and glucose transporter (Iserovich et al., 2002; Yang and Verkman, 2002; Zeuthen et al., 2016).

The CO_2_-transporting function of APQ1 is replaceable by RhAG gas channel (Ripoche et al., 2006; Endeward et al., 2008; Musa-Aziz et al., 2009). The functional overlaps between AQP1 and RhAG may explain why defective CO_2_ transport is not observed in AQP1 knockout mice (Yang et al., 2000). Interestingly, recent new findings from AQP1 knockout mice suggest that AQP1 expression enhances one’s tolerance or endurance for extreme physical activities (Xu et al., 2010; Al-Samir et al., 2016a,b). Their results somehow resonate with our findings from studying the GP.Mur phenotype, which suggest potential involvement of AQP1 in blood CO_2_ respiration and physical tolerance. Unlike the clear-cut monogenic effect of kidney AQP1 in urine concentration, the role of AQP1 in red cell functions remains to be explored.

## Author Contributions

The author confirms being the sole contributor of this work and approved it for publication.

## Conflict of Interest Statement

The author declares that the research was conducted in the absence of any commercial or financial relationships that could be construed as a potential conflict of interest.
